# The Role of Psoas Muscle in Scoliosis: A Review of the Literature

**DOI:** 10.7759/cureus.83144

**Published:** 2025-04-28

**Authors:** Zoi Fryda, Kalliroi-Antonia Passadi, Sevina Giannakou, Andriana Vasilakou, Konstantina Pouli, Panagiotis Lepetsos, Christos P Zafeiris

**Affiliations:** 1 2nd Orthopedic Department, KAT Trauma Hospital of Athens, Athens, GRC; 2 2nd Orthopedic Department, Gennimatas General Hospital, Athens, GRC; 3 Arthroscopy and Endoprosthetics Department, City Hospital Nettetal, Nettetal, DEU; 4 2nd Orthopedic Department, National and Kapodistrian University of Athens Medical School, “Konstantopouleion” Hospital, Athens, GRC; 5 2nd Orthopedic Department, Agia Sofia Children's Hospital, Athens, GRC; 6 Orthopedic Department, Athens Medical Center, Athens, GRC; 7 Orthopedics and Spine Surgery Department, Metropolitan General Hospital, Athens, GRC

**Keywords:** csa, curvature, psoas muscle, scoliosis, spine

## Abstract

Scoliosis, a lateral curvature of the spine, is influenced by various factors, including muscular imbalances. The psoas muscle, due to its anatomical positioning and function, has been hypothesized to influence scoliotic changes. The aim of this review is to evaluate the current evidence on the role of the psoas muscle in scoliosis. A search was performed using PubMed, Scopus, and Web of Science databases for studies published up to July 2024. Keywords used included “psoas” AND “scoliosis”. Inclusion criteria were studies that examined the anatomical, physiological, and clinical aspects of the psoas muscle in scoliosis. Systematic reviews, studies in languages other than English language and conference papers were excluded. Studies not specifically addressing the psoas muscle were also excluded. After the application of research methodology, 32 studies remained for analysis. The psoas muscle plays a significant role in the pathogenesis and progression of scoliosis through mechanisms involving asymmetrical activity, biomechanical imbalances, neuromuscular factors, and growth and development. The cross-sectional area (CSA) of the psoas muscle affects spinal stability, increasing the likelihood of asymmetrical loading and subsequent curvature of the spine. Moreover, it affects the outcome of scoliosis surgery. The transpsoas approach is a novel minimally invasive technique for scoliosis surgery offering reduced tissue damage, quicker recovery, and improved visualization of the disc space. Psoas-related complications of scoliosis surgery, such as psoas weakness or psoas hematoma, exist and cannot be overlooked. Randomized trials are needed to validate psoas-targeted exercises or botulinum toxin injections in the treatment of scoliosis. Future research should focus on elucidating the mechanisms by which the psoas muscle affects scoliosis and exploring comprehensive treatment approaches.

## Introduction and background

Scoliosis is a multifaceted spinal deformity that manifests as an abnormal lateral curvature of the spine, accompanied by vertebral rotation and, often, a thoracic rib hump. This condition is prevalent across various age groups and can be classified into several types, including idiopathic, congenital, neuromuscular, and syndromic scoliosis [1,2].

Idiopathic scoliosis, the most common form, affects approximately 2%-3% of the adolescent population and is further subdivided into infantile, juvenile, and adolescent idiopathic scoliosis. Infantile idiopathic scoliosis occurs in children aged 0 to three, is relatively rare, and often resolves spontaneously [3]. Juvenile idiopathic scoliosis is diagnosed in children aged four to 10 and has a higher risk of progression compared to infantile scoliosis [4]. Adolescent idiopathic scoliosis is the most common type, occurring in children aged 10-18. It accounts for approximately 80% of all idiopathic cases and often progresses rapidly during growth spurts [5,6]. Congenital scoliosis arises from vertebral malformations during fetal development, such as hemivertebrae, vertebral bar formation, or segmentation anomalies, leading to significant spinal deformities and is often associated with other organ system abnormalities, such as congenital heart defects or renal anomalies [7]. Neuromuscular scoliosis is associated with neuromuscular disorders such as cerebral palsy, muscular dystrophy, and spinal muscular atrophy. Syndromic scoliosis occurs as a symptom of genetic syndromes such as Marfan syndrome, Ehlers-Danlos syndrome, and neurofibromatosis [8]. Another type of scoliosis that affects adults and is associated with aging is degenerative scoliosis, characterized by an abnormal curvature of the spine that develops due to the degeneration of the spinal discs and facet joints [9,10].

The pathophysiology of scoliosis is complex and multifactorial, involving genetic, hormonal, neuromuscular, and biomechanical factors. Genome-wide association studies (GWAS) have pinpointed loci on chromosomes 8q12 and 17q25 linked to adolescent idiopathic scoliosis, suggesting a polygenic inheritance pattern [11]. Hormonal factors, particularly during puberty, are thought to contribute to scoliosis progression. Abnormal melatonin signaling has been implicated in idiopathic scoliosis, as melatonin influences skeletal growth and development. Additionally, growth hormone levels and estrogen metabolism may impact the growth velocity and curvature progression in scoliosis patients [12]. Conditions that affect muscle tone, motor control, and spinal stability, such as cerebral palsy and muscular dystrophy, predispose individuals to spinal curvature due to the lack of symmetrical muscular support and control. Asymmetrical growth and mechanical forces acting on the spine contribute to scoliosis development.

Common signs and symptoms of scoliosis include spinal curvature, often noticed by the patient or caregivers. Shoulder, waist or hip asymmetry, along with prominent rib hump may be present. Diagnosis depends on inspection of patient posture, shoulder height, and waist asymmetry, a positive Adam’s forward bend test, and the use of a scoliometer. Full-length standing posteroanterior and lateral x-rays of the spine are the gold standard for the diagnosis of scoliosis. The Cobb angle, measured on the posteroanterior x-ray, quantifies the degree of spinal curvature. Magnetic resonance imaging (MRI) is indicated in cases of atypical scoliosis, rapid progression, or neurological symptoms to evaluate the spinal cord and other soft tissues [13,14].

The management of scoliosis depends on the type, severity, and risk of progression of the curvature. For mild scoliosis (Cobb angle < 20^o^) with low risk of progression, regular observation and follow-up are recommended. Bracing is the primary non-surgical treatment for moderate scoliosis (Cobb angle 20-40^o^) in growing children and adolescents, aiming to prevent progression of the curve during the growth period. Bracing is effective in halting curve progression in 70%-80% of patients if worn as prescribed (typically 16-23 hours per day) [15]. Surgical intervention is indicated for severe scoliosis (Cobb angle > 40-50^o^) or in cases where conservative management fails to halt curve progression. In an attempt to correct the deformity, stabilize the spine, and prevent further progression [16].

The psoas muscle is a deep-seated muscle located in the lower lumbar region of the spine and extending through the pelvis to the femur. The psoas muscle is part of the iliopsoas group, which consists of the psoas major and the iliacus muscle [17]. Psoas major, innervated by the femoral nerve, originates from the transverse processes of the T12-L5 vertebrae and inserts into the lesser trochanter of the femur [18]. The psoas minor, present in some individuals, originates from the T12 and L1 vertebrae and inserts into the iliopubic eminence. The primary functions of the psoas major include hip flexion, trunk flexion, lumbar spine stabilization, and postural support, maintaining an upright posture by stabilizing the pelvis and lower spine. Given these functions, any imbalance or dysfunction in the psoas muscle can have a profound impact on spinal alignment and posture [19].

The psoas muscle, due to its anatomical positioning and function, has been hypothesized to influence scoliotic changes. The aim of this review is to evaluate the current evidence on the role of the psoas muscle in scoliosis.

## Review

A search was performed using PubMed, Scopus, and Web of Science databases for studies published up to July 2024. Keywords used included “psoas” AND “scoliosis”. Inclusion criteria were studies that examined the anatomical, physiological, and clinical aspects of the psoas muscle in scoliosis. Systematic reviews, studies in languages other than English language and conference papers were excluded. Studies not specifically addressing the psoas muscle were also excluded. The term “psoas muscle” in this article refers primarily to the psoas major unless otherwise specified.

**Figure 1:**
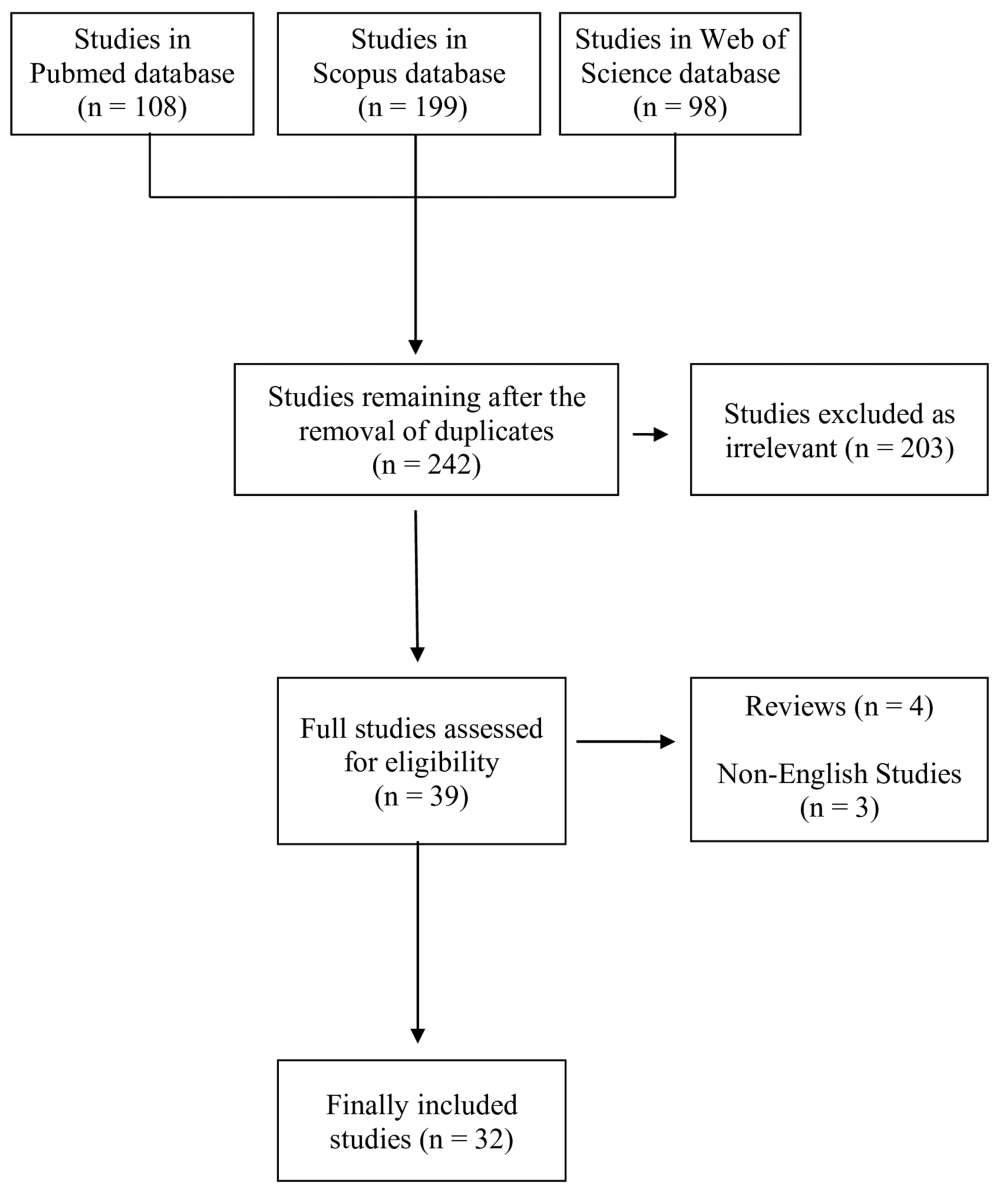
Study flowchart

Initially, 405 studies were identified after the primary search of the online databases PubMed, Scopus, and Web of Science. After checking for duplicate entries, 163 articles were excluded. Among the remaining 242 studies, 203 were rejected as irrelevant to the purpose of the study. Of the remaining studies, four review papers and three non-English studies were excluded. Finally, 32 studies remained for analysis (Figure 1).

Role of the psoas muscle in the pathogenesis of scoliosis

The psoas muscle plays a significant role in the biomechanics and stability of the lumbar spine and pelvis. Its anatomical positioning and function make it a crucial muscle for maintaining proper posture and spinal alignment [20]. Scoliosis is a complex three-dimensional deformity characterized by lateral curvature and vertebral rotation. Asymmetrical activity of the psoas muscle can lead to an imbalance in spinal and pelvic alignment. Increased tone or hypertrophy of the psoas muscle on one side can pull the spine laterally, contributing to the lateral curvature and vertebral rotation characteristic of scoliosis [21]. In healthy girls, iliopsoas muscle strength was mainly predicted by body weight, body height, and body mass index, while in girls with scoliosis, psoas muscle strength was associated with body weight and Cobb angle. However, no differences were detected in maximal voluntary isometric contraction between healthy girls and girls with scoliosis [22]. Ultrasound-guided injection of botulinum toxin in the psoas muscles in patients with adolescent idiopathic scoliosis led to significant radiological corrective changes in the frontal plane in the thoracic and lumbar spine, along with significant derotational corrective changes in the lumbar spine [21].

The role of the psoas muscle in stabilizing the lumbar spine means that any dysfunction or imbalance can alter the biomechanics of the spine. A shortened or tight psoas muscle can lead to anterior pelvic tilt and increased lumbar lordosis, which can predispose to or exacerbate scoliotic deformity. Biomechanical models and clinical observations indicate that individuals with scoliosis often exhibit tightness or contracture of the psoas muscle on the concave side of the curve. This asymmetry can cause rotational and lateral forces on the vertebral column, contributing to curve progression [20]. Bruggi et al. showed the corrective effect of the iliopsoas contraction on the scoliotic curve in patients with adolescent idiopathic scoliosis [20].

Imaging studies have revealed differences in psoas muscle size and density between scoliotic and non-scoliotic individuals. These differences are more pronounced in cases of severe scoliosis [23]. Bal and Batin, using computed tomography (CT), compared psoas muscle volumes between 34 women with scoliosis and 29 healthy women. They found that, in patients with scoliosis, psoas muscle volume was significantly greater on the convex side of the curve. When patients with scoliosis were compared to controls, the psoas muscle volume bilaterally was significantly lower. Researchers noticed that patients with idiopathic scoliosis developed atrophy of the psoas muscle on both sides of the curve, mostly on the concave side of the curve, in comparison to healthy controls [23].

Role of the cross-sectional area (CSA) of the psoas muscle

The psoas muscle is a key stabilizer of the lumbar spine. If the CSA is reduced, the spine may be less stable, increasing the likelihood of asymmetrical loading and subsequent curvature of the spine. In case of severe adolescent idiopathic scoliosis, with a mean Cobb angle of 56^o^, psoas muscles had a significantly larger CSA on the lumbar concave side [24]. In adolescents with idiopathic scoliosis, the CSA of the psoas muscle was higher on the concave side than the convex side at the level of the apical vertebra. Patients with a higher Cobb angle had statistically greater psoas muscle imbalance at the level of the apical vertebra. The CSA of the psoas muscle on both sides of patients with adolescent idiopathic scoliosis was higher than the mean CSA of healthy controls aged 16-18; however, there was no significant difference between patients with adolescent idiopathic scoliosis and healthy controls aged 10-15 [25].

On the other hand, according to Banno et al., the CSA of the psoas major muscle was not different between patients with mild scoliosis (Cobb angle < 20^ο^) and matched controls [26]. Similarly, Incesoy et al. detected no significant difference in the CSA of the psoas major among patients with Lenke V adolescent idiopathic scoliosis and controls [27]. A study by Watanabe et al. showed that CSA of the psoas muscle was not associated with the thoracolumbar curve progression in patients with adolescent idiopathic scoliosis [28].

In degenerative scoliosis, the reduction in CSA of the psoas muscle can be part of the overall degenerative process affecting spinal muscles and structures. As people age, muscle mass generally decreases (sarcopenia), which can contribute to the weakening of spinal support structures and the development of scoliosis. A recent retrospective case-control study by Chen et al. included 120 patients with degenerative scoliosis and 120 healthy controls. In patients with scoliosis, CT-calculated CSA of the psoas major was significantly smaller on the convex side than the concave side [29]. Kim et al. found that in patients with degenerative scoliosis, the difference index of CSA of the psoas muscle at the apex of curvature level was significantly higher on the convex side than that on the concave side. There was no difference in fatty infiltration of the psoas muscle and paravertebral muscles. Authors concluded that muscle hypertrophy on the convex side, rather than muscle atrophy on the concave side, may explain this asymmetry [30]. In a recent study, CSA of the psoas muscle on the concave side was significantly higher at the L2-3 and L3-4 levels in scoliotic patients. The psoas muscle on the concave side was significantly thicker with a higher fat infiltration rate [31]. Using diffusion tensor imaging, Eguchi et al. compared psoas major morphology among 12 patients with degenerative lumbar scoliosis and 10 matched controls. All patients with degenerative lumbar scoliosis had a rising psoas sign, showing that the psoas muscle, along with the lumbar nerves, shifted anteriorly together. The psoas major was significantly extended on the concave side. In 75% of patients with degenerative lumbar scoliosis, the psoas major was extended on the concave side. In the remaining 25%, the psoas major was extended on the convex side [32].

In the upper lumbar region, CSA of the psoas in patients with degenerative lumbar scoliosis was smaller than healthy controls or patients with degenerative lumbar stenosis, a finding that is not observed in the lower lumbar region. CSA of the psoas major is strongly associated with health-related quality of life in healthy individuals [33]. Comparing patients with degenerative lumbar scoliosis and patients with lumbar spinal stenosis, Yagi et al. found that the CSA of the psoas muscle was significantly smaller in the degenerative lumbar scoliosis. In these patients, CSA of the psoas muscle was correlated with sagittal spinal alignment [34]. Table 1 summarizes CSA findings from the cited studies.

**Table 1:** Comparative table summarizing the role of psoas CSA in the pathogenesis of scoliosis AIS: Adolescent idiopathic scoliosis. CSA: cross-sectional area.

Authors	Year	n	Diagnosis	Findings
Becker et al. [24]	2023	74	AIS, mean Cobb 56^o^	Psoas muscles had a significantly larger CSA on the lumbar concave side
Xu et al. [25]	2021		AIS with primary lumbar scoliosis	CSA of the psoas muscle was higher on the concave side than the convex side at the level of the apical vertebra. The CSA of the psoas muscle on both sides of patients with AIS was higher than the mean CSA of healthy controls aged 16-18
Banno et al. [26]	2017	49	Mild scoliosis, Cobb < 20^o^	CSA of the psoas muscle was not different between patients with mild scoliosis and matched controls
Incesoy et al. [27]	2023	42	AIS, Lenke V	No significant difference on the CSA of the psoas muscle among patients with Lenke V AIS and controls
Watanabe et al. [28]	2018	28	AIS, Cobb > 30^o^	CSA of the psoas muscle was not associated with the thoracolumbar curve progression in patients with AIS
Chen et al. [29]	2024	120	Degenerative scoliosis	In patients with scoliosis, CT-calculated CSA of the psoas muscle was smaller on the convex side than the concave side
Kim et al. [30]	2013	85	Degenerative scoliosis, mean Cobb 18^o^	The difference index of CSA of psoas muscle at apex of curvature level was significantly higher in convex side rather than that in concave side
Shin and Ryu [31]	2017	108	Lumbar degenerative scoliosis	CSA of the psoas muscle on the concave side was significantly higher at the L2-3 and L3-4 levels
Liu et al. [33]	2023	44	Lumbar degenerative scoliosis	CSA of the psoas in patients with degenerative lumbar scoliosis were smaller than healthy controls
Yagi et al. [34]	2016	60	Degenerative scoliosis	CSA of psoas muscle was significantly smaller in the degenerative lumbar scoliosis. In these patients, CSA of psoas muscle was correlated with sagittal spinal alignment

During periods of rapid growth, such as adolescence, the differential growth rates of the vertebrae and surrounding musculature, including the psoas muscle, can influence spinal alignment. An imbalance in the growth and development of the psoas muscle can contribute to the onset and progression of scoliosis. The psoas muscle, due to its role in hip flexion and spinal stability, may be implicated in rapid curve progression. Before skeletal maturity, there is a significant psoas muscle imbalance in patients with adolescent idiopathic scoliosis, and the imbalance is related to the severity of scoliosis. The morphology of the psoas muscle changed with the progression of scoliosis [25].

Role of the psoas muscle in the management of scoliosis

The psoas muscle, due to its critical role in hip flexion and lumbar spine stabilization, is integral to the management of scoliosis. Effective management of scoliosis often includes strategies to address muscle imbalances, improve spinal alignment, and enhance postural stability, all of which involve the psoas muscle.

Physical therapy plays a central role in managing scoliosis, particularly for mild to moderate cases. Specific stretching exercises can help lengthen the psoas muscle, reducing its pull on the lumbar spine and pelvis. Strengthening the psoas muscle can enhance its stabilizing function for the lumbar spine. According to Bruggi et al., “the unilateral contraction of the psoas muscle, aimed to obtain hypertrophy and shortening of the muscle tissue that appears to be longer than the contralateral muscle, causes an anterior flexion, or a lateral tilt of the spine towards the side of the contracting muscle, and a slight contralateral rotation of the vertebrae”. In patients with moderate adolescent idiopathic scoliosis (mean Cobb angle 20º), the combination of these effects results in three-dimensional correction of the scoliotic curve [20].

CSA of the psoas muscle has been found to affect the outcome of scoliosis surgery. In patients undergoing posterior stabilization for adolescent idiopathic scoliosis, the CSA ratio of the erector spinae muscle was correlated with the CSA ratio of the psoas major. Therefore, in these patients, the minimization of the injury to the erector spinae muscle is vital to the preservation of psoas major development [35]. Degeneration of psoas muscles affects lower instrumented vertebra screw loosening in six or more level fusion in corrective surgery for degenerative lumbar scoliosis [36]. Psoas major degeneration predisposes for screw loosening in patients with adult degenerative scoliosis [37]. A retrospective study by Han et al. suggested that the psoas major relative functional CSA after corrective surgery for degenerative lumbar scoliosis was significantly smaller in patients with last follow-up T1 pelvic angle > 20º [38]. Distal instrumentation-related problems in patients with degenerative lumbar scoliosis undergoing long-instrumented spinal fusion are not associated with relative functional CSA and the gross muscle-fat index of the psoas muscle [39]. According to Tanida et al., the decrease in lumbar lordosis and the lateral deviation of the lumbar spine following scoliosis caused a deviated course of the psoas muscle, which was spontaneously corrected by the restoration of lumbar alignment [40].

Transpsoas approach for scoliosis surgery

Transpsoas surgery, also known as the lateral lumbar interbody fusion (LLIF) or direct lateral interbody fusion (DLIF), is a minimally invasive spinal fusion technique used to address various spinal conditions, including idiopathic and degenerative scoliosis. This approach involves making a lateral incision and navigating through the psoas muscle to reach the lumbar spine, which allows for the placement of interbody spacers and fusion devices without disrupting the posterior spinal musculature, thus potentially reducing muscle damage, bleeding, and postoperative pain [32,41-44]. Table 2 summarizes the advantages and the disadvantages of the transposas approach in scoliosis surgery.

**Table 2:** Advantages and disadvantages of transpsoas approach in scoliosis surgery

Advantages	Disadvantages
1) Minimally invasive, avoids posterior muscle dissection.	1) Risk of injury to the lumbar plexus.
2) Less soft tissue and muscle disruption compared to posterior approaches.	2) Postoperative thigh numbness, hip flexor weakness due to psoas muscle manipulation.
3) Shorter hospital stay and faster recovery compared to open posterior fusion.	3) Potential for vascular injury if anterior structures are not carefully protected.
4) Less intraoperative blood loss.	4) Risk of persistent iliopsoas or groin pain.
5) Less postoperative pain.	

Clinical results of the transpsoas LLIF have been encouraging. Asad et al. reported a 13% reduction of Oswestry Disability Index (ODI) and a 100% fusion rate, among 32 patients undergoing transpsoas LLIF for degenerative scoliosis [42]. Dakwar et al. reported the results of the lateral transpsoas approach in 25 patients with adult degenerative scoliosis. A mean 23.7% improvement in ODI was observed along with an 80% rate of fusion [41]. Khajavi and Shen evaluated 21 patients with degenerative scoliosis, undergoing transpsoas LLIF. A 50% improvement in disability and a 59% pain reduction were observed in a two-year follow-up [43]. A similar small study by Takami et al. found a 100% fusion rate, with a significant improvement in disability, pain, and spinopelvic parameters and an 11.8% re-operation rate [44].

Psoas-related complications of scoliosis surgery

Scoliosis surgery, particularly procedures involving the lumbar spine, can be associated with various complications related to the psoas muscle. Due to its anatomical location adjacent to the lumbar vertebrae, surgical interventions in this region can affect the psoas muscle, leading to a range of complications. Intraoperative damage to blood vessels within or near the psoas muscle can lead to bleeding and subsequent hematoma formation. This can occur during the insertion of screws or rods and in case of violation of the intertransverse plane [45,46]. Weakness of the psoas muscle can occur due to direct muscle trauma, lumbar plexus or femoral nerve injury, or disuse atrophy due to prolonged postoperative immobilization [44]. Other psoas-related complications include psoas abscess, femoral nerve injury, and psoas syndrome.

While transpsoas surgery offers many advantages, there are specific risks and considerations associated with the procedure. Navigating through or dilating the psoas muscle poses a risk of injury to the muscle itself and the lumbar plexus, particularly the femoral nerve [32,42,44,47,48]. Neuromonitoring is often used to mitigate this risk [41]. Lumbar nerve course tracking with diffusion tensor imaging is useful for assessing patients with degenerative lumbar scoliosis before LLIF [32]. A supra-psoas shallow docking technique appeared to minimize postoperative neurological morbidity by docking on top of the psoas muscle instead of passing through it, allowing for direct visualization of the lumbosacral plexus [47]. In LLIF, a convex approach is relatively safer than an approach from the concave side, as nerves on the convex side showed a posterior shift [32]. Similarly, Myung-Hoon et al. suggested that the convex approach is safer, as the convex retroperitoneal vessels were positioned more anteriorly, whereas the ventral nerve roots lacked significant positional variations, thus providing optimal disc space access and minimizing psoas muscle injury [31]. Transpsoas approach from the concave side may endanger a potential injury to the lumbar artery, in female patients with lumbar scoliosis and a Cobb angle > 14.5^ο^ [46]. A multi-institutional, retrospective study found that the rate of neurological complications was significantly higher in the convex approach compared with the concave approach (26.9% versus 4.0%) [49]. On the contrary, according to Mai et al., due to anatomic variations, “an approach toward the concave side may have a more predictable course for surrounding anatomy” [48]. Moreover, “approaching the intervertebral space from the patient's left may reduce the risk of encountering critical vascular structures” [48]. Patients may experience temporary thigh pain or weakness due to manipulation of the psoas muscle and associated nerves during surgery [32]. The lateral approach may not be suitable for all levels of the lumbar spine, particularly L5-S1, due to the anatomical positioning of the iliac crest [41].

## Conclusions

The psoas muscle plays a significant role in the pathogenesis and progression of scoliosis through mechanisms involving asymmetrical activity, biomechanical imbalances, neuromuscular factors, and growth and development, and may significantly influence the successful outcome of the scoliosis surgery. Maintaining its strength and balance is crucial for spinal stability and posture. The transpsoas approach is useful for lumbar interbody fusion in patients with degenerative lumbar scoliosis. Randomized trials are needed to validate psoas-targeted exercises or botulinum toxin injections in the treatment of scoliosis. Future research should focus on elucidating the mechanisms by which the psoas muscle affects scoliosis and exploring comprehensive treatment approaches.
